# Mismatch Repair Protein Msh2 Is Necessary for Macronuclear Stability and Micronuclear Division in *Tetrahymena thermophila*

**DOI:** 10.3390/ijms241310559

**Published:** 2023-06-23

**Authors:** Lin Wang, Yuhuan Xue, Sitong Yang, Tao Bo, Jing Xu, Wei Wang

**Affiliations:** 1 Key Laboratory of Chemical Biology and Molecular Engineering of Ministry of Education, Institute of Biotechnology, Shanxi University, Taiyuan 030006, China; 201913002004@email.sxu.edu.cn (L.W.); 202223002021@email.sxu.edu.cn (Y.X.); yangsitong77@163.com (S.Y.); botao@sxu.edu.cn (T.B.); 2 Shanxi Key Laboratory of Biotechnology, Taiyuan 030006, China; 3 School of Life Science, Shanxi University, Taiyuan 030006, China

**Keywords:** *Tetrahymena thermophila*, mismatch repair protein, macronucleus, micronucleus

## Abstract

Mismatch repair (MMR) is a conserved mechanism that is primarily responsible for the repair of DNA mismatches during DNA replication. Msh2 forms MutS heterodimer complexes that initiate the MMR in eukaryotes. The function of Msh2 is less clear under different chromatin structures. *Tetrahymena thermophila* contains a transcriptionally active macronucleus (MAC) and a transcriptionally silent micronucleus (MIC) in the same cytoplasm. Msh2 is localized in the MAC and MIC during vegetative growth. Msh2 is localized in the perinuclear region around the MIC and forms a spindle-like structure as the MIC divides. During the early conjugation stage, Msh2 is localized in the MIC and disappears from the parental MAC. Msh2 is localized in the new MAC and new MIC during the late conjugation stage. Msh2 also forms a spindle-like structure with a meiotic MIC and mitotic gametic nucleus. *MSH2* knockdown inhibits the division of MAC and MIC during vegetative growth and affects cellular proliferation. *MSH2* knockdown mutants are sensitive to cisplatin treatment. *MSH2* knockdown also affects micronuclear meiosis and gametogenesis during sexual development. Furthermore, Msh2 interacts with MMR-dependent and MMR-independent factors. Therefore, Msh2 is necessary for macronuclear stability, as well as micronuclear mitosis and meiosis in *Tetrahymena*.

## 1. Introduction

Mismatch repair (MMR) is a conserved mechanism that primarily repairs mismatches that occur during DNA replication. These mismatches include base–base mismatches and insertion/deletion loops [1]. The MMR system is an important mechanism for maintaining the stability of a genome. The highly conserved nature of MMR proteins from bacteria and yeast to humans emphasizes the significant role of this protein family [2]. The MutHLS MMR pathway in *Escherichia coli* has been defined and reconstructed in vitro. MMR begins with the highly conserved MutS protein, which recognizes mismatched bases and activates the endonuclease activity of MutH with MutL [3]. MutS and MutL function as homodimers in prokaryotes and as heterodimeric complexes in eukaryotes. The MutSα (Msh2–Msh6) heterodimer complex binds to the mismatch site and is the initiating step in the eukaryotic MMR mechanism [4].

Deficiencies in these MMR mechanisms lead to various cancers [5]. In addition, the lack of MMR-related proteins in cancer cells is a major source of cellular resistance to chemotherapeutic drugs [2,6,7,8]. The synergistic effect of Msh2 and circRNA circLIFR enhances the therapeutic effect of the chemotherapeutic agent cisplatin (DDP) on bladder cancer cells [9]. hMsh2-deficient cells are resistant to DDP-induced apoptosis and DNA damage signals [10]. Although Msh2-deficient mouse embryonic fibroblasts have shown a resistance to DDP, Msh2^G674A^ tumors reduce their proliferation capacity after a DDP induction [11]. In eukaryotic cells, the genome is packaged into chromatin. The chromatin context of the genome facilitates and regulates DNA repair [12]. The correct assembly and disassembly of nucleosomes are necessary for maintaining genomic and epigenetic stability. During DNA replication, base mismatches occur after the replication-dependent disassembly of chromatin. The cell ensures that these mismatches have been repaired before the reassembly of nucleosomes on the newly replicated DNA. The histone assembly mechanism and MMR mechanism regulate each other [12]. Human MutSα inhibits the polymerization of the Caf-1- and Asf1a-dependent H3-H4 tetramers on DNA [13,14]. An *MSH2* deficiency in primary mouse embryonic fibroblasts significantly increases chromosomal aneuploidy, centrosome amplification, defective mitotic spindle organization, and unequal chromosomal segregation [15].

MutS, MutL, MutH, and helicase UvrD are necessary for partial homologous recombination in *E. coli* [16]. The hMsh2-hMsh6 heterodimer in human cells also plays an important role in the rejection of heterodimeric DNA [17]. Msh4 and Msh5 form heterodimers that bind and stabilize DNA strand exchange intermediates, thereby promoting the formation of class I meiotic crossover [18,19]. Knocking out *MSH4* or *MSH5* in *T. thermophila* leads to a reduction in chiasma during meiosis [20]. It has been shown that Msh2 is involved in homologous recombination [17,21]. Msh2-Msh3 promotes DNA end excision during recombination [21]. The anti-recombination function of Msh2-Msh6 limits telomere extension through the homology-directed alternative lengthening of telomeres repair mechanisms [22]. Msh2-Msh3 binds Holliday junctions and acts on the resolution of these Holliday junctions by interacting directly with Slx4 [23]. The function of Msh2 is less clear under different chromatin structures.

At present, the function of the MMR complex has not been fully elucidated because of the inherent complex and transient interaction between MMR proteins and DNA substrates [24]. *Tetrahymena thermophila* has two functionally distinct nuclei, namely, a germline micronucleus (MIC) and somatic macronucleus (MAC) [25]. MAC chromosomes are polyploid, approximately 45-fold, and are divided by amitosis and degraded during sexual reproduction [26]. The MIC is diploid and transcriptionally inactive during vegetative growth [27]. The MIC undergoes meiosis and produces gametic nuclei during sexual reproduction [25]. The genome distribution in the nuclear division of *Tetrahymena* has three different patterns, including micronuclear mitosis, meiosis, and macronuclear amitosis [28]. During meiosis, a programmed double-strand break (DSB) occurs to initiate homologous recombination. The meiotic recombination in *Tetrahymena* is dependent on Spo11 and DSB [29]. The meiotic process in *Tetrahymena* is characterized by a micronuclear expansion to twice the cell length in the meiotic prophase [30]. MIC expansion is triggered by the formation of DSB [31]. The MMR proteins Msh4-Msh5 and Mlh1-Mlh3 in yeast are involved in the Class I recombination pathway [20]. In addition, the homologous proteins Msh4-Msh5 in *Tetrahymena* are involved in meiotic recombination [20]. Although the expression profiles of *TMLH1* and *MLH3* suggest the function of the proteins during meiosis, the knockdown of these proteins does not result in significant meiotic defects in *Tetrahymena* [32]. The mutation in human *MSH2* causes the majority of hereditary nonpolyposis colorectal cancer [33]. Here, we explored the localization and function of Msh2 in *T. thermophila*. Msh2 was dynamically localized in the MAC and MIC. *MSH2* knockdown mutants affected nuclear stability and they were sensitive to treatment with cisplatin. In addition, Msh2 interacted with the MMR-dependent factor Msh6 or Msh6L3 and with MMR-independent factors such as motor proteins and the proteins involved in the redox processes.

## 2. Results

### 2.1. Characterization of MutS Proteins in T. thermophila

**Figure 1:**
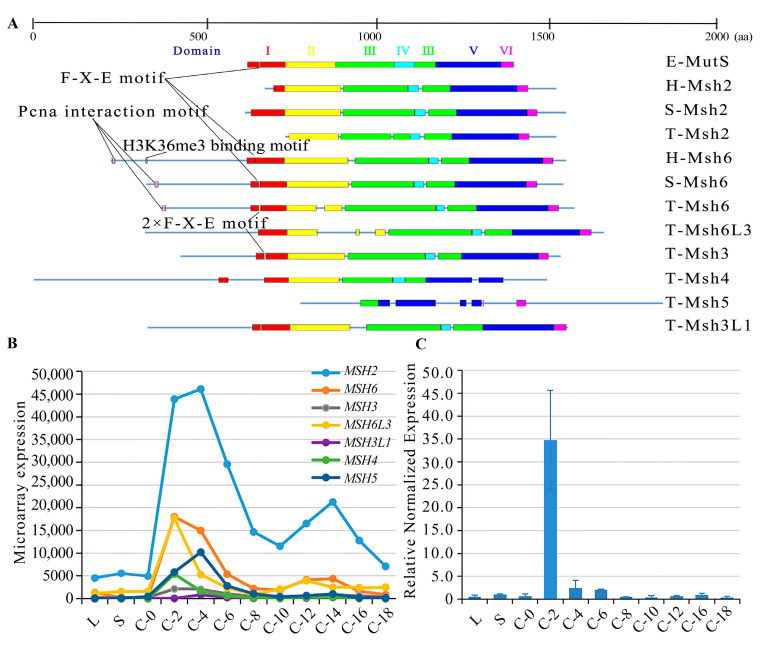
MutS homologs in *Tetrahymena*. (**A**) Comparison of amino acid sequences of *E. coli* MutS proteins, human and yeast Msh2 and Msh6 proteins, and all MutS homologs in *T. thermophila*. E represents *E. coli*; H represents human; S represents *S. cerevisiae*; and T represents *T. thermophila*; (**B**) Expression profiles of MutS homologs in *T. thermophila* from the TetraFGD (http://tfgd.ihb.ac.cn/, accessed on 30 August 2022); Y-axis indicates the microarray expression profiles of MutS homologs. (**C**) Relative expression profiles of the *MSH2* gene at different development stages. Y-axis indicates the relative normalized expression of the *MSH2*. L, vegetative proliferation; S, starvation; For conjugation, equal volumes of B2086 and CU428 cells were mixed, and samples were collected at 0, 2, 4, 6, 8, 10, 12, 14, 16, and 18 h after mixing (referred to as C-0, C-2, C-4, C-6, C-8, C-10, C-12, C-14, C-16, and C-18).

MutS proteins are diverse in eukaryotes. Six MutS proteins, Msh1-6, have been identified in the *S. cerevisiae* genome [34]. Among these, Msh1, which maintains the stability of the mitochondrial genome, has not yet been identified in mammals [35]. MutS homologs in mammals include Msh2-6 [36]. The Msh6 paralog Msh7 has been found in plants [37]. Msh4 and Msh5 do not participate in MMR and they are involved in meiotic homologous recombination in budding yeast [18,38], humans [19], and *Tetrahymena* [20]. Here, seven MutS homologous proteins were identified in *T. thermophila* (Table S1). These MutS homologs have six conserved structural domains (Figure 1A). Msh6 and Msh3 exhibited a duplication of the Phe-X-Glu motif in domain I. This motif acted directly on the mismatched bases in *E. coli* [39]. Furthermore, the N-terminal end of Msh6 interacted with PCNA. Msh2 showed the lowest similarity to the other MutS homologs (Table S2), probably because Msh2 lacks domain I, which is a mismatch recognition domain [39,40]. *MSH2* showed the highest expression level compared to the other MutS genes (Figure 1B). A phylogenetic analysis showed that Msh2 and Msh6 are conserved within the species (Figure S1). *T. thermophila* was at the bottom of the eukaryotic evolutionary tree, which is consistent with the commonly held view that ciliates and metazoans diverge or almost diverge at the bottom of the eukaryotic phylogenetic tree [41]. Among the MutS homologs, Msh6 clustered with Msh6L1, Msh3 (TTHERM_00426230), Msh6L3 (TTHERM_00150000), and Msh3L1 (TTHERM_00142230). Msh2 diverged early from the other *T. thermophila* MutS proteins.

### 2.2. Msh2-3HA Was Localized in the MAC and MIC during Vegetative Growth

**Figure 2:**
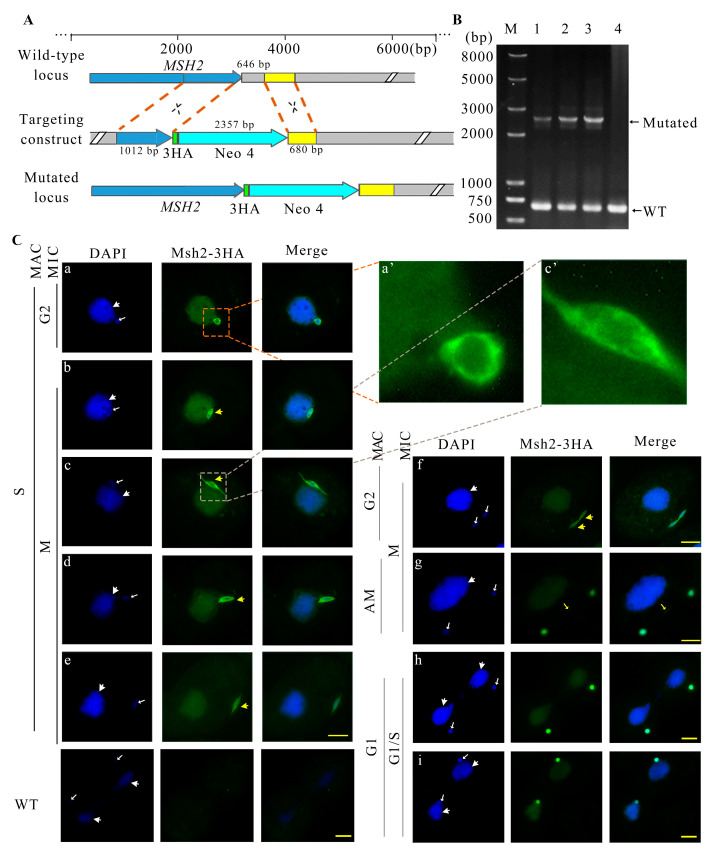
Localization of Msh2-3HA to the amitotic MAC and mitotic MIC during the vegetative proliferation of *Tetrahymena*. (**A**) Schematic representation for generating recombinant Msh2-3HA mutants in *T. thermophila*. The blue box in the wild-type locus indicates *MSH2*; the grey box refers to downstream sequences of *MSH2*; and the yellow box indicates homologous arm. The blue box in the mutation locus indicates the *MSH2* gene. The 646 bp grey box is replaced by 3HA and Neo4 cassette. (**B**) The identification of Msh2-3HA-B2086 and Msh2-3HA-CU428 mutants. Concentration on paromomycin that PCR was performed at 8 mg/mL. M indicates marker; 1 and 2 indicate Msh2-3HA-B2086 mutant cell line; 3 indicates Msh2-3HA-Cu428 mutant cell line; and 4 indicates wild-type cells. WT and mutant loci were amplified by PCR, the mutated locus, 2357 bp; the wild-type locus, 646 bp; (**C**) Immunofluorescence localization of Msh2-3HA. In the G2 phase of the MIC, the Msh2-3HA is more strongly localized in the perinuclear than in the nuclear, showing a circular localization around the MIC (**a**). The spindle-like structure is indicated by yellow arrows with large heads (**b**–**f**). A yellow arrow with a small head indicates the localization of Msh2-3HA in the non-chromatin part of the nucleus during mitosis (**g**). In the G1/S phase of MIC, the localization of Msh2-3HA on the micronucleus shows a solid circle shape (**h**,**i**).The white arrows with the large head indicate the position of the MAC and the white arrow with the small head indicates the MIC. DAPI stains the nuclei and Msh2-3HA is localized by indirect immunofluorescence via HA tagging (green). The enlarged part (**a**’,**c**’)is five times larger than the corresponding part (**a**,**c**). The scale bar is 10 μm.

Msh2 was predicted to be localized in the nucleus with Euk-mPLoc 2.0. The predicted nuclear localization signal for Msh2 was 341 IDMEKARRDNEYQVSSKFSPTLAELAKQMKQI 372. A HA-tag was constructed at the C-terminus of the *MSH2* gene via homologous recombination (Figure 2A). Msh2-3HA mutants were created (Figure 2B). Msh2-3HA was evenly distributed in the MAC throughout the vegetative growth stage (Figure 2C). At the beginning of micronuclear mitosis, the elongated MIC was parallel to the long axis of the cell and it was half buried on the surface of the MAC; moreover, Msh2-3HA formed a spindle-like structure (Figure 2C(b)). Next, when the MIC was pulled away from the MAC, Msh2-3HA was localized to the “thin thread” nucleus (Figure 2C(c)). Then, the “thin thread” shortened and disappeared, leaving one end of the MIC toward the cell cortex and the other end connected to the MAC. Msh2-3HA showed a strong distribution in the perinuclear region (Figure 2C(d)). α-Tubulin and Msh2-3HA were partially co-localized in the perinuclear region of the spindle-like MIC (Figure S2a). Subsequently, the MIC left the MAC and attached to the cell cortex, and Msh2-3HA was uniformly localized to the MIC (Figure 2C(e)). Before the onset of the micronuclear anaphase, the MIC was stretched as a dumbbell shape (Figure 2C(f,g)) and Msh2-3HA was evenly localized in the circular and intermediate sections (Figure 2C(g)). α-Tubulin and Msh2-3HA were co-localized in the intermediate section (Figure S2b). The divided MICs remained attached to the MAC after cytoplasmic division, during which Msh2-3HA remained evenly localized to the MIC (Figure 2C(h,i)). Upon the completion of mitosis, Msh2-3HA was more strongly localized in the perinuclear region than the nucleus, showing a circular localization around the MIC (Figure 2C(a)). During pre-starvation, Msh2-3HA was uniformly localized on the MIC, but after 24 h of starvation, the localization of Msh2-3HA on the MIC migrated toward the perinuclear region (Figure S3A). Msh2-3HA was uniformly localized to the nucleus after detergent treatment and the localization of the perinuclear area disappeared (Figure S3B). The results implied Msh2 functions during micronuclear DNA replication and chromosomal disjunction.

### 2.3. Msh2 Localized in the MIC and New MAC during Sexual Development

**Figure 3:**
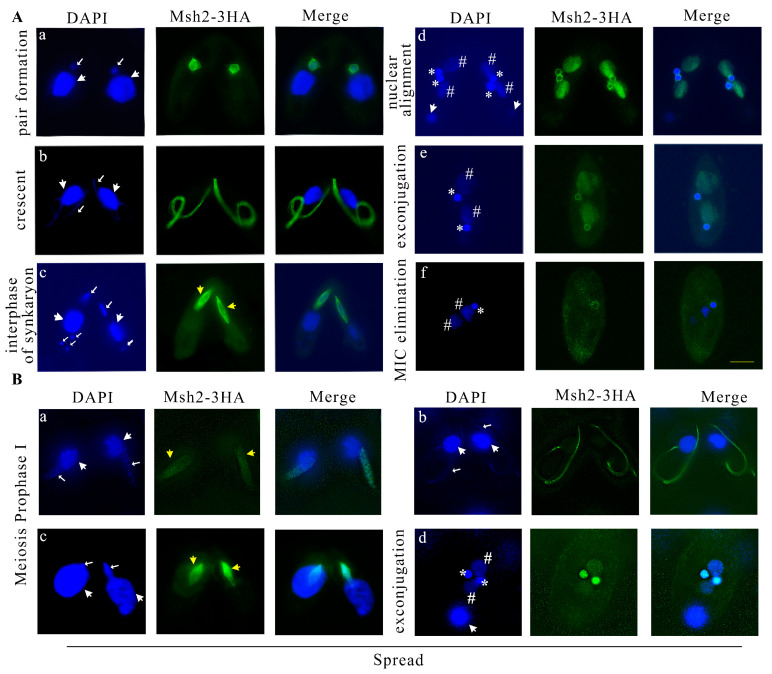
Msh2-3HA is localized in the MIC and newly developed MAC during the conjugation of *Tetrahymena*. (**A**) Immunofluorescence localization of Msh2-3HA during conjugation. During pair formation, Msh2-3HA localized to the MIC (**a**). During the micronuclear crescent stage, Msh2-3HA was localized on the elongated MIC (**b**). During pronuclear mitosis, Msh2-3HA formed a spindle-like structure (**c**). Msh2-3HA was localized in the new MACs as well as in the perinucleus of the new MICs during nuclear alignment and exconjugation (**d**,**e**). Msh2-3HA localized to the new MIC during MIC elimination (**f**). (**B**) Localization of Msh2-3HA on chromatin in spread cells. Msh2-3HA was localized to the chromatin during MIC meiosis prophase Ι after the detergent treatment (**a**–**c**). During exconjugation, Msh2-3HA evenly localized on the new MIC and new MAC (**d**). DAPI stains the nuclei and the localization signal of Msh2-3HA is localized by indirect immunofluorescence via HA tagging (green). The white arrows with the large head indicate the position of the MAC and the white arrow with the small head indicates the MIC. * indicates the new MIC and # indicates the new MAC. The spindle-like structure is indicated by yellow arrows with large heads. The scale bar is 10 μm.

During sexual development, the parental MAC transcribes but does not replicate, whereas the MIC replicates and performs meiosis and pronuclear mitosis. After sexual development was initiated, Msh2-3HA localized to the MIC and no signal in the parental MAC was observed (Figure 3A(a)). During the micronuclear crescent stage, Msh2-3HA was localized on the elongated MIC (Figure 3A(b)) and co-localized with α-tubulin (Figure S2c). During pronuclear mitosis, Msh2-3HA formed a spindle-like structure and it was more significantly and strongly localized in the perinuclear region than the nucleus (Figure 3A(c)). However, the parental degraded meiotic products had no staining signal (Figure 3A(c)). Msh2-3HA was also co-localized with α-tubulin in the perinuclear region during pronuclear mitosis (Figure S2d). During late sexual development, the zygotic nucleus performed mitotic division and formed two new MACs and two new MICs. Msh2-3HA was localized on the new MACs and MICs. However, in contrast to the evenly distributed localization on the new MACs, the localization on the new MICs was uneven, showing a stronger signal in the perinuclear region (Figure 3A(d)). This staining was maintained until pair separation (Figure 3A(e)). Finally, one of the new MICs was eliminated. Msh2-3HA remained on the new MIC and disappeared on the new MAC (Figure 3A(f)). The results strongly showed that Msh2 is involved in DNA replication and mitosis, but does not play a role in the transcription of the parental MAC.

The interaction between MMR proteins and chromatin has been demonstrated in eukaryotes [12,42,43,44]. In examining the pattern of interaction between Msh2 and meiotic chromatin, soluble proteins were removed from cells using detergent spreading [45]. Msh2-3HA was strongly localized to the chromatin during MIC meiosis prophase Ι after the detergent treatment (Figure 3B(a–c)). After the pairing was separated, Msh2-3HA was evenly localized on the new MIC and MAC, and after the detergent treatment, its localization in the perinuclear region was lost (Figure 3B(d)).

### 2.4. MSH2 Knocking Down Affected the Division of Nuclei during Vegetative Growing Stage

**Figure 4:**
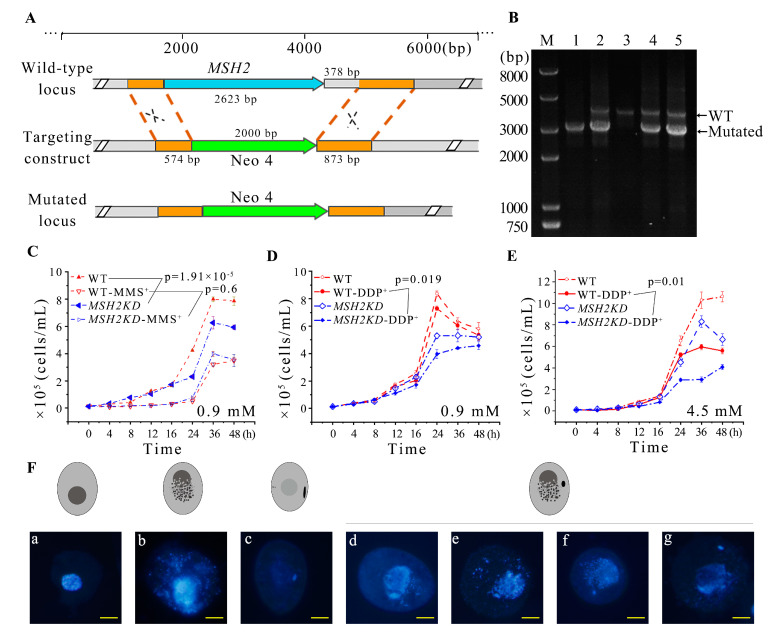
Knockdown of *MSH2* affected nuclear division and cellular proliferation during vegetative growth in *Tetrahymena*. (**A**) Schematic representation for generating recombinant *MSH2KD* mutants. The orange box indicates the homologous sequence used for homologous substitution. The blue arrow in the wild-type locus indicates *MSH2. MSH2* and a 378 bp downstream region, were replaced by the Neo4 cassette; (**B**) Identification of *MSH2KD*-B2086 and *MSH2KD*-CU428 mutants. M indicates marker; 1 and 2 indicate *MSH2KD*-Cu428 mutant cell line; 3 indicates wild-type cells; and 4 and 5 indicate *MSH2KD*-B2086 mutant cell line. WT and mutant loci were amplified by PCR, the mutated locus with a fragment length of 3040 bp, the wild-type locus, 4046 bp; (**C**–**E**) Vegetative growth curves of wild-type cells and *MSH2KD* mutant cells before or after treatment with different DNA damage reagents at 30 °C for 48 h. (**C**) 0.9 mM MMS; (**D**) 0.9 mM DDP; and (**E**) 4.5 mM DDP. *Tetrahymena* exhibited more severe DNA damage after DDP was added (*p* < 0.05) (**D**,**E**). The cell concentration is 0.125 × 10^5^ cells/mL; (**F**) Abnormal morphology of nuclear division in *MSH2KD* cells after temperature-induced synchronized division, with a model of cell development labeled above the morphology of nuclear division. The MIC was degraded alone (**a**) or degraded with the MAC (**b**). Weak MAC staining (**c**). The MAC was fragmented and dispersed in the cell (**d**–**g**).

**Figure 5:**
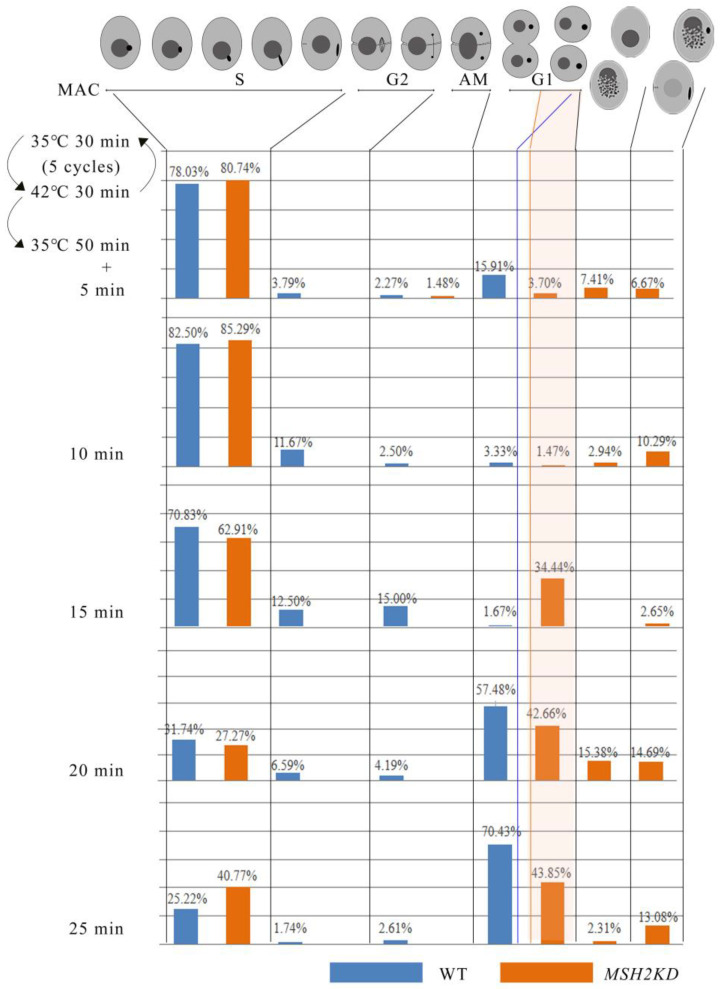
Knockdown of *MSH2* affected nuclear division during vegetative proliferation in *T. thermophila*. Nuclear development statistics of the MAC of the *MSH2KD* mutant cell line and wild-type cells after synchronization during vegetative proliferation. The number of cells counted was greater than 120 cells, for each time point, for each cell line. The topmost part of the diagram shows a diagram of the cell development model.

To explore the function of *MSH2*, it was knocked out from the somatic genome (Figure 4A). Partial-knockout *MSH2KD* mutants were obtained (Figure 4B). The proliferation of the *MSH2KD* mutants was significantly decreased (*p* < 0.05, Figure 4C). Nuclear development was observed in the *MSH2KD* cells after temperature-induced synchronized division to explore the cause of the diminished proliferative capacity of the *MSH2KD* cells. After synchronization, the macronuclear development of the *MSH2KD* cells was stalled in the S phase (Figure 5). In some mutants, the MAC staining was weaker (Figure 4F(c)). In other mutants, the MAC was fragmented and dispersed in the cell (Figure 4F(b,d–g)). The MIC was also degraded alone (Figure 4F(a)) or degraded with the MAC (Figure 4F(b)). In some cells, the MIC that had stopped division returned to the G1/S phase (Figure S4) after breaking away from its attachment to the MAC. Therefore, the knockdown of *MSH2* affected the normal progression of macronuclear amitosis and micronuclear mitosis during the vegetative proliferation of *Tetrahymena*, which, in turn, reduced the proliferation capacity. In addition, the sensitivity of the *MSH2KD* mutants to MMS was consistent with that of their wild type (*p* > 0.05), whereas the mutants were more sensitive to DDP (*p* < 0.05, Figure 4C–E).

### 2.5. Knocking Down MSH2 Affected Sexual Development 

**Figure 6:**
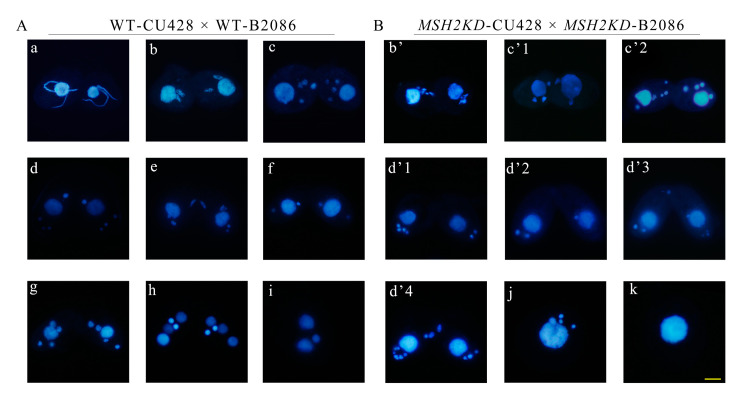
Knockdown of *MSH2* affects nuclear division during conjugation in *Tetrahymena*. The representative nuclear morphology of wild-type (**A**) and *MSH2KD* mutant (**B**) cells was shown during conjugation stage. **a**: crescent elongates; **b**: the first prezygotic division (Meiosis Ι); **c**: second prezygotic division (Meiosis II); **d**: MIC “selection”; **e**: gamete nucleus mitosis; **f**: first postzygotic division; **g**: second postzygotic division; **h**: nuclear alignment; **i**: MIC apoptosis; **b**’, **c**’1, **c**’2, and **d**’1–**d**’4 indicate the abnormal cell phenotypes of the corresponding periods; and **j** and **k** stand for single cells with abnormalities. The MICs of *MSH2KD* cells were fragmented in that they did not form proper spindle-like structures (**b**’) as in wild-type cells (**b**). The number and morphology of MICs in *MSH2KD* cells were abnormal (**c**’1 and **c**’2) compared to wild-type cells (**c**). The MICs of the *MSH2KD* cells accumulated at the posterior of the cells without selected MICs at the anterior of the cells (**d**’1–**d**’3), as in the wild-type cells (**d**). More than 3 MICs at the posterior of the cell (**d**’4). DAPI stains the nuclei blue. Scale bar is 10 μm. DAPI stains the nuclei.

The expression of *MSH2* was the highest at 2 h of conjugation (Figure 1C, Table S5). During early conjugation (2–4 h), the mating of the *MSH2KD* mutants was normal and similar to that of the WT cells. However, the MICs had an abnormal morphology with abnormal nuclear division (Figure 6A). The MICs of the *MSH2KD* cells were fragmented at the end of the first prezygotic division (Meiosis Ι) and they did not form proper spindle-like structures (Figure 6B(b’)) as they did in the wild-type cells (Figure 6A(b)) [26]. The number and morphology of the second prezygotic division (Meiosis II) products in the *MSH2KD* cells were abnormal (Figure 6B(c’1,c’2)). At 6–7 h of conjugation, 43.67–60.76% of the *MSH2KD* paired cells were stalled during the MIC “selection” stage and 80% of these stalled cells had an abnormal nuclear developmental phenotype (Figure S5). The meiotic products of the *MSH2KD* cells accumulated at the posterior of the cells and failed to select the functional pronuclei (Figure 6B(d’1–d’4)). At 8–12 h of conjugation, only 4.9–15.23% of *MSH2KD* mating cells (61.54–44.90% of wild-type mated cells) exhibited parental macronuclear apoptosis, new MAC formation, and pair separation. At 24 h of conjugation, only 16% of *MSH2KD* mating cells (41.6% of the wild-type cells) normally finished sexual development and 42.38% of these cells were abnormal (Figure S5). The abnormal MICs (Figure 6B(j)) were degraded and mutant cells without MICs were observed (Figure 6B(k)). The mutant phenotype was partially rescued by mating with the WT cells. After 24 h of conjugation, 37.25% of the rescued mating cells finished sexual development (Figure S5).

### 2.6. Conditional Knockdown of MSH2 Affected Sexual Development during Conjugation

To further explore the stage-specific function of Msh2, conditional knockdown *msh2i* mutants were constructed (Figure S6A). *MSH2* was conditionally knocked down by artificially controlling the time and dose of Cd^2+^ addition. The proliferation of the *msh2i* mutants was significantly decreased (*p* < 0.05) when the expression level of *MSH2* was downregulated under Cd^2+^ induction (Figure S6B,C, Table S6). Then, the knockdown of *MSH2* in the *msh2i* mutants was induced by adding Cd^2+^ at 12 h of starvation. Early on, at 2–4 h of conjugation, the mating mutants with Cd^2+^ induction showed abnormal MICs. The MICs had an abnormal morphology with a shorter crescent structure and unevenly distributed chromatin (Figure S6D(a’1,a’2)). The MICs were fragmented at the end of meiosis Ι and they did not form proper structures (Figure S6D(b’1–5)) like they did in the wild-type cells (Figure S6D(b)). After meiosis II, the number and morphology of MICs were abnormal (Figure S6D(c’1,c’2)). At 7 h of conjugation, 29.32% of the *msh2i* paired cells were in the MIC “selection” stage and 60.71% of them showed an abnormal nuclear development phenotype (Figures S6D(d’) and S7). At 24 h of conjugation, 17.49% of the cells finished sexual development and formed an exconjugant with two MAC cells and one MIC cell (58.02% for WT, Figure S7). Without Cd^2+^ induction, 34.16% of the paired cells were in the MIC “selection” stage and 16.13% of them were abnormal at 7 h of conjugation; 34.99% of the cells finished sexual development and formed an exconjugant with two MAC cells and one MIC cell at 24 h of conjugation (Figure S7). The abnormal MICs (Figure S6D(g)) were degraded and mutant cells without MICs were observed (Figure S6D(h)). The results confirmed that *MSH2* is involved in meiosis and is necessary for sexual development during conjugation.

### 2.7. Msh2 Interacted with MMR-Dependent and MMR-Independent Factors 

**Figure 7:**
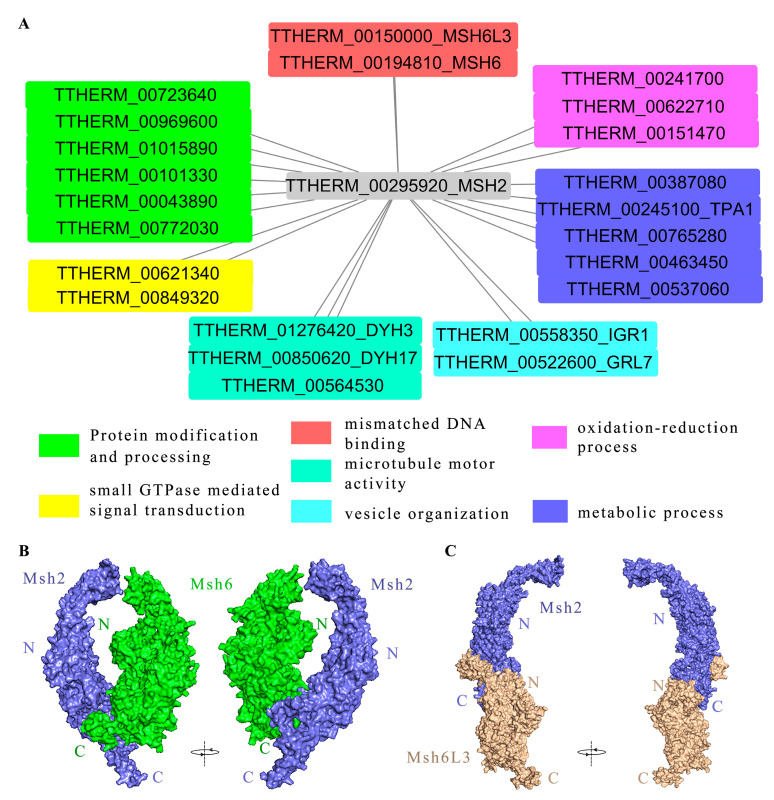
Interacting proteins of Msh2 are involved in a variety of cellular processes. (**A**) Protein interaction network map of Msh2 at 3 h conjugation; the HA tag at the C-terminus of the Msh2-3HA was used to immunoprecipitate the Msh2 interaction proteins in *T. thermophila*. Then, the interaction proteins were identified using mass spectrometry analysis. MaxQuant was used to analyze mass spectrometry data. (**B**,**C**) Protein–protein docking of Msh2 and Msh6 (**B**), as well as Msh2 and Msh6L3 (**C**).

We used the HA-tag at the C-terminus of the Msh2 protein to immunoprecipitate the interaction protein of Msh2 in *T. thermophila*, followed by a mass spectrometry analysis to identify the interaction proteins [46]. The Msh2-3HA protein and the protein physically bound to Msh2 were captured together with the HA antibody. A series of washes were necessary to elute the unbound proteins [46]. The threshold value of iBAQ WT/iBAQ Msh2HA was 0.05 (Table S4). Based on the protein interaction network diagram (Figure 7A), the following proteins interacted with Msh2 at 3 h of conjugation, including MMR-dependent factors such as the DNA-binding protein Msh6 (TTHERM_00194810) and Msh6L3 (TTHERM_00150000), which are involved in the MMR mechanism. Msh6 forms a heterodimer complex with Msh2, which binds mismatch sites and serves as an important gene for initiating MMR in eukaryotes [4]. Msh6L3 is a MutS homolog specifically found in *T. thermophila*. We further observed the interaction model of Msh2 and Msh6 using protein–protein docking. Msh2 interacted with Msh6 via the C-terminus of the proteins (Figure 7B). However, the C-terminus of Msh2 interacted with the N-terminal end of Msh6L3 to form a heterodimer (Figure 7C). In addition, MMR-independent factors were also identified, such as Dynein Heavy chain Dyh3 (TTHERM_01276420), Dyh17 (TTHERM_00850620), and kinesin motor catalytic domain protein (TTHERM_00564530), which are motor proteins. DYH3 and DYH17 are axonemal dynein heavy chain genes of *Tetrahymena* that generate ciliary and flagellar forces by sliding double microtubules [47]. TTHERM_00622710, TTHERM_00241700, and TTHERM_00151470 possess oxidoreductase activity, which participates in redox processes. TTHERM_00043890 and TTHERM_00772030 with olichyl-diphosphooligosaccharide- protein glycotransferase activity on endoplasmic reticulum membranes are involved in protein glycosylation. TTHERM_00723640, TTHERM_00969600, and TTHERM_01015890 have protein tyrosine kinase activity. TTHERM_00101330 can bind unfolded proteins and participate in protein folding. TTHERM_00849320 is involved in small GTPase-mediated signal transduction. TTHERM_00621340 is involved in the regulation of Rab GTPase activity. The results indicated that Msh2 interacted with MMR-dependent and MMR-independent factors and that it is involved in different signal pathways.

## 3. Discussion

MMR proteins are a family of post-replication repair proteins that are highly conserved from bacteria to humans [2]. As an MMR initiator, MutS recognizes mismatch sites [3]. Seven MutS proteins have been identified in *T. thermophila*; Msh2 (TTHERM_00295920) is the homolog of yeast and human Msh2, and the expression of *MSH2* is the highest compared to the other MutS proteins in *Tetrahymena*. The MutSα complex differentiates into a heterodimeric complex in the early branching protozoan world. The structural domain of MutS is conserved among species. However, some of the structural domains of MutS in *Tetrahymena* have been lost or duplicated. Msh2 in *T. thermophila* lacks domain I. The MutS protein in *E. coli* functions as a homodimer, with subunit A domain Ι and subunit B domain IV functioning in the recognition of DNA-containing mismatches [48], whereas subunit A domain IV and subunit B domain Ι are not involved in DNA recognition. By contrast, MutS serves as a heterodimer Msh2–Msh6 complex in eukaryotes. Msh6 interacts directly with DNA [49]. Msh6 and Msh3 in *T. thermophila* exhibit the duplication of the Phe-X-Glu motif in domain I, which could enhance the ability of Msh6-binding DNA.

Msh2 in HeLa MR cells localizes in the cytoplasm and nucleus. In the presence of N-methyl-N9-nitro-N-nitrosoguanidine, Msh2 translocates from the cytoplasm into the nucleus [50]. In *T. thermophila*, Msh2 migrated from the perinuclear to the intra-nuclear region during mitosis. Msh2 colocalization with α-tubulin formed a spindle-like perinuclear region in mitotic MICs, which may be associated with MIC stretching. In addition, Msh2 formed thin threads at the end of the stretching MIC, which could be related to MIC migration. During the late mitotic M-phase, the intermediate section did not contain chromatin or DNA and it was later cut off and shed into the cytoplasm, where it was eventually taken up by the cell [26]. The localization of Msh2 in the intermediate section and co-localization with α-tubulin imply that Msh2 functions in chromatin segregation. The mitosis of the MIC failed to proceed normally after the *MSH2* knockdown and it could not enter the late M phase. Msh2 was uniformly localized to the MAC throughout MAC amitosis. After the *MSH2* knockdown, the MAC failed to develop into G2 and AM phases. In some cells, the MAC was fragmented. *MSH2* knockdown affected macronuclear amitosis and micronuclear mitosis in *Tetrahymena*, which led to abnormal nuclear division and inhibited cellular proliferation.

The MIC stretches approximately 50-fold during prophase I of meiosis, forming a “crescent” structure, where homologous meiotic recombination and DSBs occur [28]. The localization of Msh2 on the crescent stage indicated that Msh2 could be associated with meiotic homologous recombination and DSBs. Msh2 was tightly bound on the chromosome, providing the necessary conditions for Msh2 to participate in meiotic homologous recombination progress. Msh2 colocalization with α-tubulin during meiosis implies that Msh2 was related to chromosomal disjunction during meiosis. The nuclear development of *MSH2KD* mutant cells was abnormal during meiosis, thereby causing some cells to stagnate during nuclear selection. The spindle-like localization and co-localization of α-tubulin with Msh2 on the gamete nucleus might be related to chromosomal disjunction during gametic mitosis. Immediately after the postzygotic completion of two mitotic divisions, the two diploid macronuclear anlagen began DNA synthesis, and they were approximately octoploid by the time they completed conjugation. By contrast, the two diploid MICs did not undergo extensive DNA replication [51]. After the postzygotic completion of two mitotic divisions until MIC elimination, the localization of Msh2 in the perinuclear region of the MICs was more pronounced than that in the nuclei, but Msh2 was tightly bound to chromatin in the nuclei rather than the perinuclear region. On the new MAC, Msh2 was evenly distributed. Therefore, Msh2 is involved in DNA replication in new MACs and nuclear remodeling in new MICs. In addition, Msh2 plays an important role in nuclear division during vegetative growth and sexual reproduction in *Tetrahymena*. Msh2 was involved in all three different genomic distribution patterns, including MIC mitosis, MIC meiosis, and MAC amitosis in *Tetrahymena*.

A prerequisite for the function of DNA damage repair proteins is their localization in the nucleus [52]. The predicted nuclear localization signal for Msh2 in yeast cells is PDKKLKL, which targets Msh2 localization in the nucleus [53]. Although GFP-hMsh2 was localized to the nucleus, hMsh2 formed a complex with importin α/β3 in an in vitro interaction assay and hMsh2 did not contain a classical nuclear localization signal [54]. hExo1, hMlh1, and hMsh2 formed a complex and entered the nucleus together [54]. The *Tetrahymena* Msh2 had a weak predicted nuclear localization signal; however, Msh2 was strongly localized in the MAC and MIC. The nuclear pore complex of the MAC in *T. thermophila* only allowed for the passage of proteins up to approximately 50 kDa via free diffusion or passive transport, whereas the nuclear pore complex of the MIC had a smaller pore size (only 10–20 kDa) [55]. The Msh2 was approximately 93.1 kDa and was presumably transported into the nucleus via active transport. Based on nuclear localization signals, there may be other mechanisms by which Msh2 is transported into the nucleus in *Tetrahymena*, such as co-entry after complexing with another repair-related protein containing a nuclear localization sequence in the cytoplasm [52]. Msh2 deficiency leads to cellular resistance against DNA damage reagents in mammalian cells [2,6,7,8] and the resistance of cells to DDP is increased when hMlh1 or hMsh6 is deficient [56]. Msh2^−/−^ mouse embryonic fibroblasts did not respond to cisplatin treatment; however, the growth of Msh2*^G674A^* missense mutation tumors was significantly suppressed after cisplatin treatment [11]. Similarly, the *MSH2KD* mutant is sensitive to DDP in *Tetrahymena*.

The MutSα (Msh2-Msh6) and MutSβ (Msh2-Msh3) complexes have different substrate specificities and play different roles in mismatch repair. The formation of the complex of Msh2 and Msh6 occurs in the cytoplasm. The preformed MutSα complex is translocated from the cytoplasm into the nucleus [50]. We found that Msh2 interacts with Msh6 (TTHERM_00194810) and Msh6L3 (TTHERM_00150000) in *Tetrahymena*. The protein–protein docking of Msh2 and Msh6 revealed that they were able to form a heterodimer with a C-terminal interaction domain, which is the same as that reported in *E. coli* and humans [39,57]. Although Msh6L3 has a domain VI at the C-terminus, Msh2 interacts with the N-terminus of Msh6L3 to form a heterodimer. In addition, Msh2 also interacts with MMR-independent proteins, such as redox processes, protein modification and processing, microtubule molecular motors, and the metabolic pathway.

Collectively, Msh2 was dynamically localized in the MAC and MIC and it formed a spindle-like structure around the MIC. The *MSH2* knockdown affected the macronuclear stability and micronuclear mitosis during vegetative growth. *MSH2* is necessary for sexual development. Msh2 interacted with MMR-dependent and MMR-independent factors, and they were involved in different signal pathways in *Tetrahymena*.

## 4. Materials and Methods

### 4.1. Culture, Starvation, and Pairing of T. thermophila

*T. thermophila* B2086 (mating type II) and CU428 (mating type VII) were obtained from the National Tetrahymena Stock Center (Cornell University, Ithaca, NY, USA). *T. thermophila* cells were grown in 1× Super Proteose Peptone medium at a constant temperature of 30 °C [58]. The cells cultured to the logarithmic phase were collected and resuspended in 10 mM Tris-HCl to maintain the cell concentration at 2.5–3 × 10^5^ cells/mL and they were incubated at 30 °C for 18–24 h [59]. The *T. thermophila* cells of different mating types were mixed in equal number of cells at 30 °C.

### 4.2. Identification of MutS Homologous Proteins

A blast analysis of the Msh2 amino acid sequence of *Saccharomyces cerevisiae* was conducted on the *Tetrahymena* Genome Database (TGD, https://tet.ciliate.org/, accessed on 1 August 2022), and proteins with an E value of <10^−6^ in the Blast results were considered to be MutS homologs in *T. thermophila*. The amino acid sequences of the proteins were compared for similarity using DNAMAN, and the percentage of similarity between each of the two sequences was determined. After obtaining multiple sequence comparisons using T-Coffee (https://tcoffee.crg.eu/apps/tcoffee/references.html, accessed on 23 September 2022), the protein structure domain comparison results were embellished and mapped using Snapgene (https://www.snapgene.com/, accessed on 17 December 2022) and Photoshop 2020 (Adobe). A phylogenetic analysis of the Msh2, Msh6, and MutS homologs in *T. thermophila* was performed using Mega-X, with 1000 bootstraps to construct a neighbor-joining tree. The expression profiles of the relevant genes are available on the TetraFGD (http://tfgd.ihb.ac.cn/, accessed on 30 August 2022).

### 4.3. Construct of Msh2-3HA Mutant Cell Line

The detailed steps for the construction of the plasmids were conducted as previously described [60]. In brief, a 1012 bp sequence at the C-terminus of the *MSH2* gene was amplified via PCR using *MSH2*-5F/R primers (the primer sequences used in this study are listed in Table S3). A 680 bp fragment downstream of *MSH2* was amplified using *MSH2*-3F/R primers. The PCR products were cloned into pGM-19T. After sequencing, the recombinant plasmid pGM-19T-5 and pHA-Neo4 vector were digested with *Sac* Ι and *Not* Ι. The purified 5’ homologous arm and pHA-Neo4 were ligated into pHA-Neo4-5. pHA-Neo4-5 and pGM-19T-3 were digested with *Xho* I and *Kpn* I. The target fragments were ligated into pNeo4-*MSH2*-3HA. The transformation used biolistic bombardment, as described previously (GJ-1000 [SCIENTZ, Ningbo, China]) [60]. After resistance screening using paromomycin, the mutant cell lines were identified via PCR using the primer *MSH2*-3HA-Identify-F/R.

### 4.4. Immunofluorescent Localization Analysis

The cells were fixed using 2% paraformaldehyde (PFA). The fixed cells were centrifuged at 300× *g* for 1 min and then 9.6 mL of PBS and 400 μL of 10% (*v*/*v*) TritonX-100 were added. Subsequently, the cells were centrifuged at 300× *g* for 1 min and the supernatant was discarded. The cells were washed three times with pre-cooled PBS. Next, 30–50 μL of the cells was evenly dispersed on coverslips coated with poly-L-lysine (Sigma-Aldrich, MO, USA) and left to dry at room temperature. After washing with PBST (PBS with 0.1% Tween-20), the coverslips were incubated with blocking buffer (3% [*v*/*v*] BSA calf serum and 10% [*v*/*v*] goat serum dissolved in PBST) for 1 h. Then, the coverslips were incubated with anti-HA (1:500, Cell Signaling Technology, Danvers, MA, USA) or anti-α-tubulin (1:200, Sigma, Santa Clara, CA, USA) for 2 h. The coverslips were subsequently incubated with FITC-conjugated anti-rabbit IgG antibody (1:1000, Millipore, Darmstadt, Germany) for 1 h. Finally, the coverslips were incubated in 1 μg/mL of DAPI for 5-10 min [45]. The slides were observed using a DeltaVision Elite microscope (Applied Precision/GE Healthcare) or a fluorescent microscope (BX51, OLYMPUS, Tokyo, Japan).

For the cytological detection of chromatin-associated proteins, the cells were treated with enhanced detergent spreading to determine if the target protein was tightly bound to the chromatin, and no localization signal would be detected for a non-chromatin tightly bound protein [45]. The experimental approach was established as previously described [45]. After taking 5 mL of a cell sample, 450 μL of 10% (*v*/*v*) TritonX-100 and 50 μL of 37% formaldehyde were added and the sample was placed on ice for 25–30 min. Then, 450 μL of 37% formaldehyde was added to the sample and it was placed at room temperature for 5 min. The cells were collected and resuspended in 500 μL of fixative solution (4% PFA, 3.5% sucrose). Next, 50 μL of the cells was evenly dispersed on coverslips coated with poly-L-lysine. Antibody incubation and cell observation were subsequently performed as in immunofluorescence localization experiments.

### 4.5. Construction of MSH2 Knockout Mutants

The 574 bp 5’ flanking sequence of *MSH2* was amplified via PCR using the primer *MSH2*-KO-5F/R. In addition, the 873 bp downstream 3’ flanking sequence of *MSH2* was amplified using the primer *MSH2*-KO-3F/R. The pKO-Neo4 vector was linearized by *BamH* I and ligated with the 5’ flanking sequence fragment using the Hieff Clone^®^ Plus One Step Cloning Kit (Yeasen, Shanghai, China). In addition, ligation and transformation were performed to obtain pKO-Neo4-5, with the 5’ flanking sequence constructed on the pKO-Neo4 vector. pKO-Neo4-5 was cleaved using *Xho* I and ligated using the 3’ flanking sequence of *MSH2*. The obtained pKO-Neo4-*MSH2* was linearized and transformed into *Tetrahymena* cells via biolistic bombardment. The mutants were identified via PCR using the primer KO-*MSH2*-iF/KO-*MSH2*-iR after paromomycin gradient screening.

### 4.6. Synchronization of Cell Division

The cell division synchronization of *T. thermophila* was performed in accordance with the synchronization steps of *T. pyriform* [61]. In brief, 0.1–0.5 × 10^5^ cells/mL were incubated at 35 °C for 30 min and quickly placed in a water bath at 42 °C for 30 min, which was used as a cycle. After 3–5 cycles, the cells were immediately placed in a 35 °C water bath. After 50–60 min, 80% of the individuals entered the division. At this point, the samples were collected at 5 min intervals. The cells were fixed quickly by adding 2% PFA or 0.37% formaldehyde. The fixed cells were stored at 4 °C for immunofluorescence localization or nuclear development observation.

### 4.7. Nuclear Development

In total, 3–3.5 × 10^5^ cells/mL were collected at different developmental stages. Ten microliters of 37% formaldehyde solution was added to 1 mL of the sample to fix the cells. Then, 10 μL of the cells was collected and 1 μL of 1 μg/mL DAPI was added and mixed. The cells were observed using a fluorescence microscope (BX51, OLYMPUS, Japan).

### 4.8. Construction of MSH2 Conditional Knockdown Mutants

The 470 bp forward (S1) and reverse sequences (S2) (1676-2146, cDNA) of *MSH2* were amplified using the primers interfer-*MSH2*-3’F/R and interfer-*MSH2*-5’F/R, respectively. The S1 and S2 fragments were cloned into the interfering intermediate vector pIF-NRP [62]. The enzymes used for the 3’ S1 and 5’ S2 sequences included *Pst* I/*Sma* I and *Pme* I/*BamH* I, respectively. The intermediate vector was digested using *Pst* I and *BamH* I. The sample was divided into two aliquots, one of which was denatured at 99 °C for 5 min and then annealed at room temperature or on ice to induce the formation of hairpin structures. After it was verified that S1 and S2 could form hairpin structures, *NRP* on the “pIF-NRP” vector was replaced with Neo5 by using the enzymes *BamH* I and *Xho* I. Furthermore, the pIF-Neo5-*MSH2* recombinant plasmid was obtained. The recombinant plasmid was fragmented by double digestion with *Not* I and *Xho* I before biolistic transformation. The *msh2i* mutant cell line was obtained via paromomycin gradient screening. The expression of *MSH2* in the mutant cells was detected via qRT-PCR using the primer *msh2i*-iden-F1/R1.

### 4.9. qRT-PCR Analysis

The total RNA was extracted by adding 1 mL of lysis solution (TRIeasy™ Total RNA Extraction Reagent, Yeasen, Shanghai, China) to the cell samples (approximately 1 × 10^6^ cells per sample). A two-step triple pre-mix kit, MonScript™ RTIII Super Mix with dsDNase (Two-Step, Monad, Suzhou, China), was used to remove genomic DNA contamination from the total RNA and synthesize the first-strand cDNA. The reaction system was prepared using a pre-mixed solution for real-time PCR amplification (HieffTM qPCR SYBR Green Master Mix, Yeasen, Shanghai, China). The expression of *MSH2* in the cells was detected via qRT-PCR using the primer *msh2i*-iden-F1/R1, and the internal reference was 17SrRNA.

### 4.10. Co-Immunoprecipitation and Mass Spectrometry

In total, 3.5 × 10^7^ cells were collected. The immunoprecipitation procedure was in accordance with the instruction of the Pierce™ HA-Labeled Magnetic IP/Co-IP Kit (ThermoFisher, Waltham, MA, USA). Protein peptide samples were digested using protein endonuclease Trypsin and then analyzed via LCMSMS (nanoLC-QE). Tandem mass spectra were obtained using a QE mass spectrometer based on the principle of higher-energy collisional dissociation. MaxQuant, a quantitative proteomic analysis software, was used to analyze large mass spectrometry data [63]. Wild-type cell lysates, where there was no HA tag at the C-terminus of Msh2, were also captured by HA antibody and subjected to mass spectrometry, with the protein obtained being used as a blank control in subsequent analyses and deducted from the experimental group. Based on the iBAQ algorithm, the protein expression in a sample was determined, which is approximately equal to the absolute protein concentration [64]. The iBAQ intensity was used to identify the proteins that interacted with the tagged proteins by screening for differential proteins in the experimental and control groups. Proteins with an iBAQ WT/iBAQ Msh2-3HA ratio less than or equal to 0.05 were defined as those with a specific interaction with Msh2 in the cell. The TGD was used to search and classify these screened proteins in accordance with the biological processes in which they were involved. Cytoscape was used to generate an interaction network map of the screened proteins [65].

### 4.11. Protein–Protein Docking

The protein structure file.pdb was obtained by submitting the amino acid sequence of the protein of interest at Phyre 2 (http://www.sbg.bio.ic.ac.uk/phyre2/html/page.cgi?id=index, accessed on 13 February 2021) [66]. Subsequently, protein–protein docking was performed using the ClusPro (https://cluspro.org/login.php, accessed on 17 December 2022) server. The protein docking results were observed using PyMOL (https://www.pymol.org/2/, accessed on 22 December 2022), and the interactions of interest were exported and then optimized using PhotoShop 2020.

## Data Availability

All relevant data are within the paper and its additional files. The data used to support the findings of this study are available upon reasonable request.

## Supplementary Materials

The following supporting information can be downloaded at: https://www.mdpi.com/article/10.3390/ijms241310559/s1.
