# Supplementary material for: Mismatch Repair Protein Msh2 Is Necessary for Macronuclear Stability and Micronuclear Division in Tetrahymena thermophila
Source: Int J Mol Sci. 2023 Jun 23;24(13):10559. doi: 10.3390/ijms241310559 (PMC10342086; doi:10.3390/ijms241310559)
Supplement: Supplementary file 1 [file ijms-24-10559-s001.zip › ijms-Msh2-Supplementary File1(1).pdf]

Supplementary Information

Table S1. MutS protein in *T.thermophila.*

Sequences producing significant alignments:		Score (bits)	E Value
TTHERM_00295920	**Msh2** DNA mismatch repair MutS family DNA-binding domain	462	1×10^{-130}
TTHERM_00150000	DNA mismatch repair MutS family DNA-binding domain	199	8×10^{-51}
TTHERM_00194810	**Msh6** MutS domain III protein	196	6×10^{-50}
TTHERM_00426230	**Msh3** DNA mismatch repair MutS family ATPase domain	177	5×10^{-44}
TTHERM_00142230	MutS domain III protein	174	3×10^{-43}
TTHERM_00857890	MutS domain II family protein	96	2×10^{-19}
TTHERM_00763040	MutS domain V protein	70	7×10^{-12}

Table S2. Similarity comparison of MutS homologs of *T.thermophila.*

	Msh2 (00295920)	Msh6 (00194810)	Msh6L3 (00150000)	Msh3/6L1 (00426230)	Msh3L1 (00142230)	Msh4 (00857890)
Msh6 (00194810)	14.89%					
Msh6L3 (00150000)	13.00%	23.79%				
Msh3/6L1 (00426230)	15.62%	34.07%	21.39%			
Msh3L1 (00142230)	12.34%	27.06%	23.02%	25.61%		
Msh4 (00857890)	8.58%	11.13%	13.35%	10.73%	12.27%	
Msh5 (00763040)	11.01%	11.85%	10.87%	10.23%	12.93%	10.92%

Table S3. Primer sequences used in the study

Primer name	Primer sequences
MSH2-5F	GAGCTCAACTCAGCCACTCGTCCATATGTC
MSH2-5R	GCGGCCGCAGATTTTAAAGCAGAAATAATTTTTTATTTTA
MSH2-3F	CTCGAGTCATATTTTAAATATATTTTTTTTGGGAG
MSH2-3R	GGTACCAACTTAATTTATAGTTATAAAATAAATAAATAG
MSH2-MPF	AAAACCAAAGCTCGGATTAAAATTTTG
MSH2-MPR	CAAAATTTTAATCCGAGCTTTGGTTTT
MSH2-KO-5F	CTAGAACTAGTGGATCCATAATGTATTTTATTAACTTAATGATTGGC
MSH2-KO-5R	GGTACCCGGGGGATCGAGAACCTAGATTTATTTATAATTGTATG
MSH2-KO-3F	CGATACCGTCGACCTCGATCATATTTTAAATATATTTTTTTTGGGAG
MSH2-KO-3R	CCGGGCCCCCCCTCGAGCAGAGTAATTCATAGGTTATCAAATACAAAT
interfer-*MSH2*-3'F	CTGCAGTTATTAGATAAGTTGCCATATGCGTTC
interfer-*MSH2*-3'R	CCCGGGCTAAAGTTTCTGCAACAAACAAACCAT
interfer-*MSH2*-5'F	GGATCCTTATTAGATAAGTTGCCATATGCGTTC
interfer-*MSH2*-5'R	GTTTAAACCTAAAGTTTCTGCAACAAACAAACCAT
MSH2-3HA-Identify-F	GTTGTTGATCTTGCTTCTTCCTATAATG
KO-*MSH2*-iF	TAAACCTTAATTTAGAATATTTATTAGGC
KO-*MSH2*-iR	GGAGAAGTACTAAAATGATTTTGAATA
MSH2-3HA-Identify-R	AAGTTCCTCCTAAGATGTGAATAGTG
msh2i-iden-F1	GACATTAATGTGAAAGAAAAGGATAAG
msh2i -iden-R1	TAGACAGTTATAAATCACCTACAATAC
17S-F	CCTGGGAAGGTACGGGTAAT
17S-R	AAGGTTCACCAGACCATTCG

Table S4. CO-IP-MS data for Msh2-3HA-interacting proteins

Gene Model Identifier	iBAQ MSH2_HA	iBAQ WT	iBAQ WT/iBAQ MSH2_HA
TTHERM_00241700	1206.9	0	0
TTHERM_00765280	952.43	0	0
TTHERM_00564530	49.613	0	0
TTHERM_00558350	2812.2	0	0
TTHERM_00723640	40.276	0	0
TTHERM_00151470	2542.9	0	0
TTHERM_00522600	544.41	0	0
TTHERM_01276420	19.225	0	0
TTHERM_01015890	12.907	0	0
TTHERM_00101330	579.14	0	0
TTHERM_00772030	319.3	0	0
TTHERM_00043890	450.46	0	0
TTHERM_00621340	867.74	0	0
TTHERM_00463450	583.79	0	0
TTHERM_00849320	2393.5	0	0
TTHERM_00387080	21.114	0	0
TTHERM_00622710	1214.4	0	0
TTHERM_00245100	98.361	0	0
TTHERM_00969600	204.98	0	0
TTHERM_00295920	306550	289.87	0.000945588
TTHERM_00850620	38162	346.68	0.00908443
TTHERM_00194810	94985	1504.7	0.015841449
TTHERM_00150000	104330	3084.8	0.029567718
**TTHERM_00537060**	**2318.8**	**110.39**	**0.047606521**
TTHERM_00600480	347.4	36.242	0.104323546
TTHERM_00773310	24029	3200.7	0.133201548
TTHERM_000088159	5947.4	844.97	0.142073847
...	...	...	...
TTHERM_00648930	325.41	8961.6	27.53941182
TTHERM_00992830	5264.5	2100800	399.0502422
TTHERM_00151488	93.05	52126	560.1934444

The threshold value of iBAQ WT/iBAQ MSH2_HA is 0.05.

Table S5. qRT-PCR results for relative expression profiles of the *MSH2* gene

Sample	Expression	Expression SEM	Mean Cq	Cq SEM
Lh	0.4211	0.44712	33.77	1.53024
S0	1	0.0924	33.3	0.09657
C0	0.63151	0.40618	34.72	0.92725
C2	34.65898	10.92061	28.15	0.44249
C4	2.31211	1.72942	27.94	1.01445
C6	1.93963	0.15007	27.73	0.10442
C8	0.39387	0.03013	35.22	0
C10	0.36307	0.40396	35.88	0.43469
C12	0.59112	0.11139	33.08	0.18339
C16	0.86975	0.28795	33.02	0.47214
C18	0.12501	0.17756	35.84	2.04689

Table S6. qRT-PCR results for relative expression of *MSH2* in *msh2i* cells

Sample	Expression	Expression SEM	Mean Cq	Cq SEM
WT	1	0.09484	33.15	0.09462
i-*MSH2*-B2086	0.53937	0.03729	34.18	0.08807
i-*MSH2*-CU428	0.35041	0.04271	35.26	0.17408

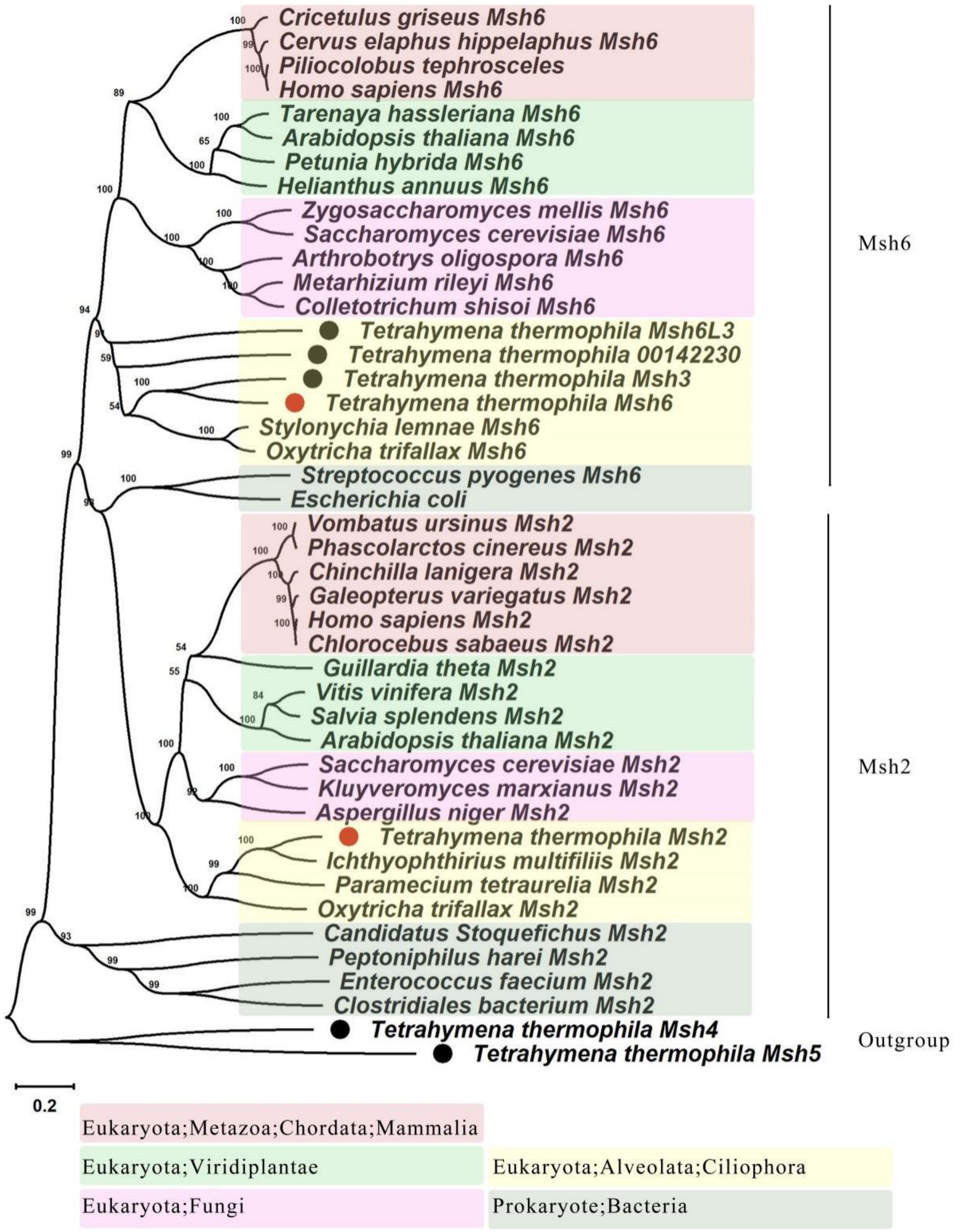

Supplementary Figure S1. Phylogenetic tree based on amino acid sequences of Msh2 and Msh6 from different species. Scale bar indicates genetic distance.

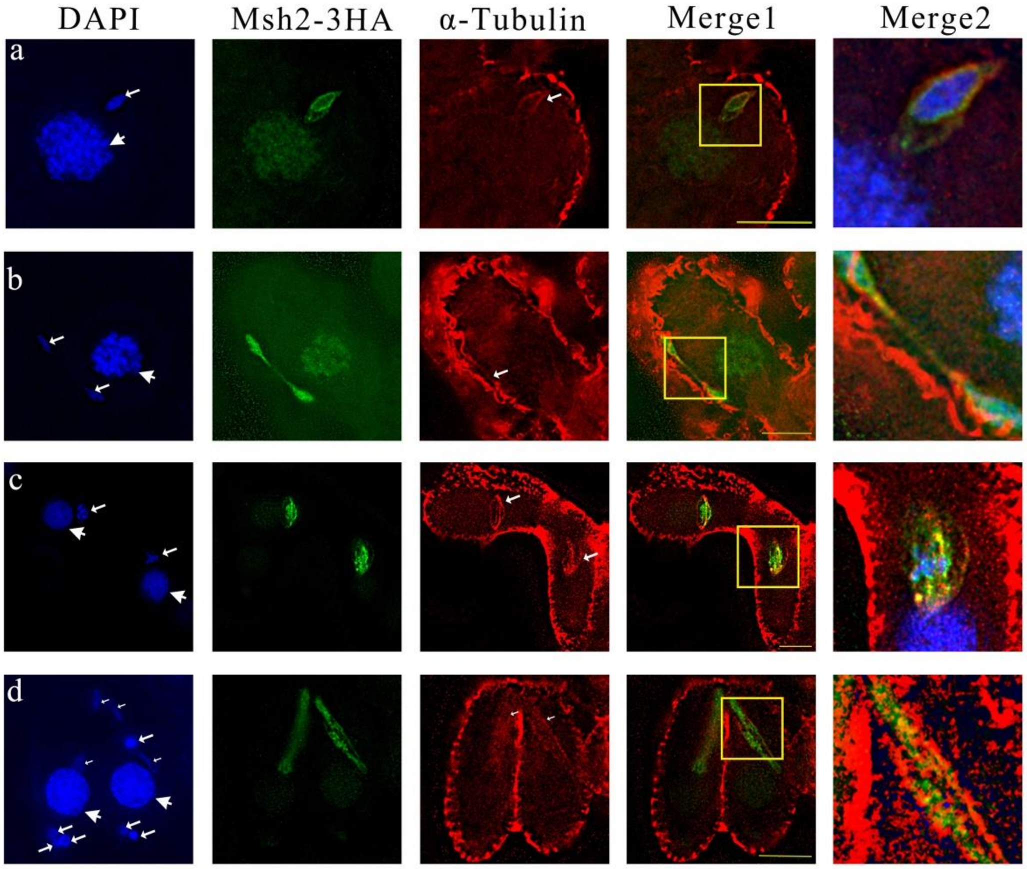

Supplementary Figure S2. Co-localization of Msh2-3HA and α-tubulin during vegetative proliferation and conjugation of *Tetrahymena*. The arrow with the larger head indicates the position of the MAC, and the arrow with the smaller head indicates the position of the MIC. Merge2 is a triple magnification of the yellow box in Merge1, incorporating DAPI, Msh2-3HA and α-tubulin. The scale bar is 10 μm.

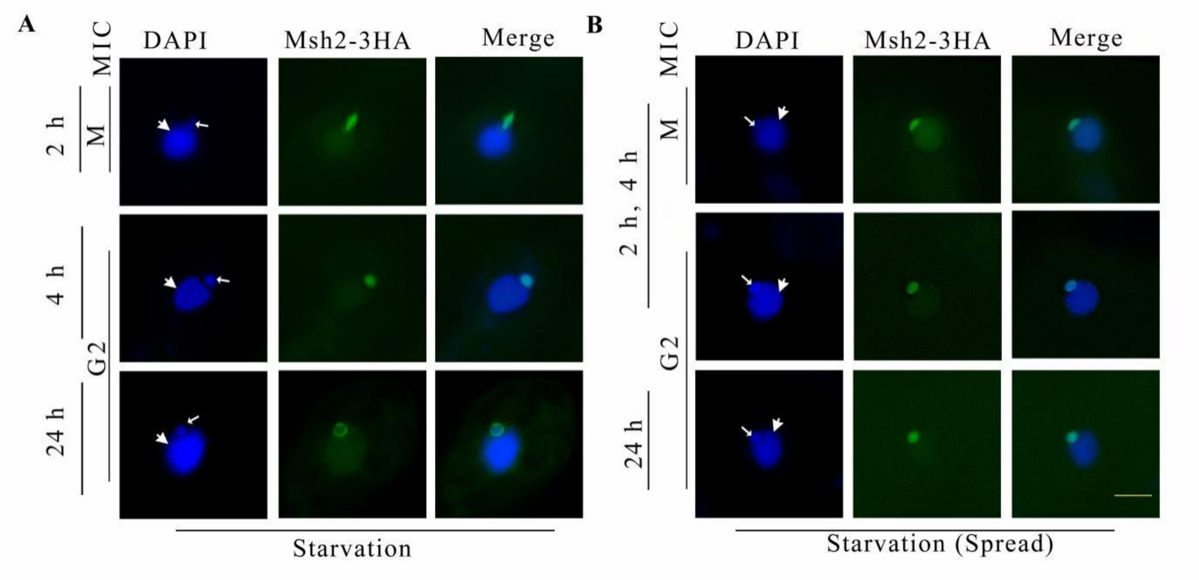

Figure S3. Msh2-3HA bound tightly to MIC and MAC chromatin during starvation. (**A**) Immunofluorescence localization of Msh2-3HA; (**B**) Localization of Msh2-3HA on chromatin in spread cells. DAPI stains the nuclei blue, and the localization signal of Msh 2-3HA is localized by indirect immunofluorescence via HA tagging (green). The arrow with the larger head indicates the position of the MAC, and the arrow with the smaller head indicates the position of MIC. The scale bar is 10 μm.

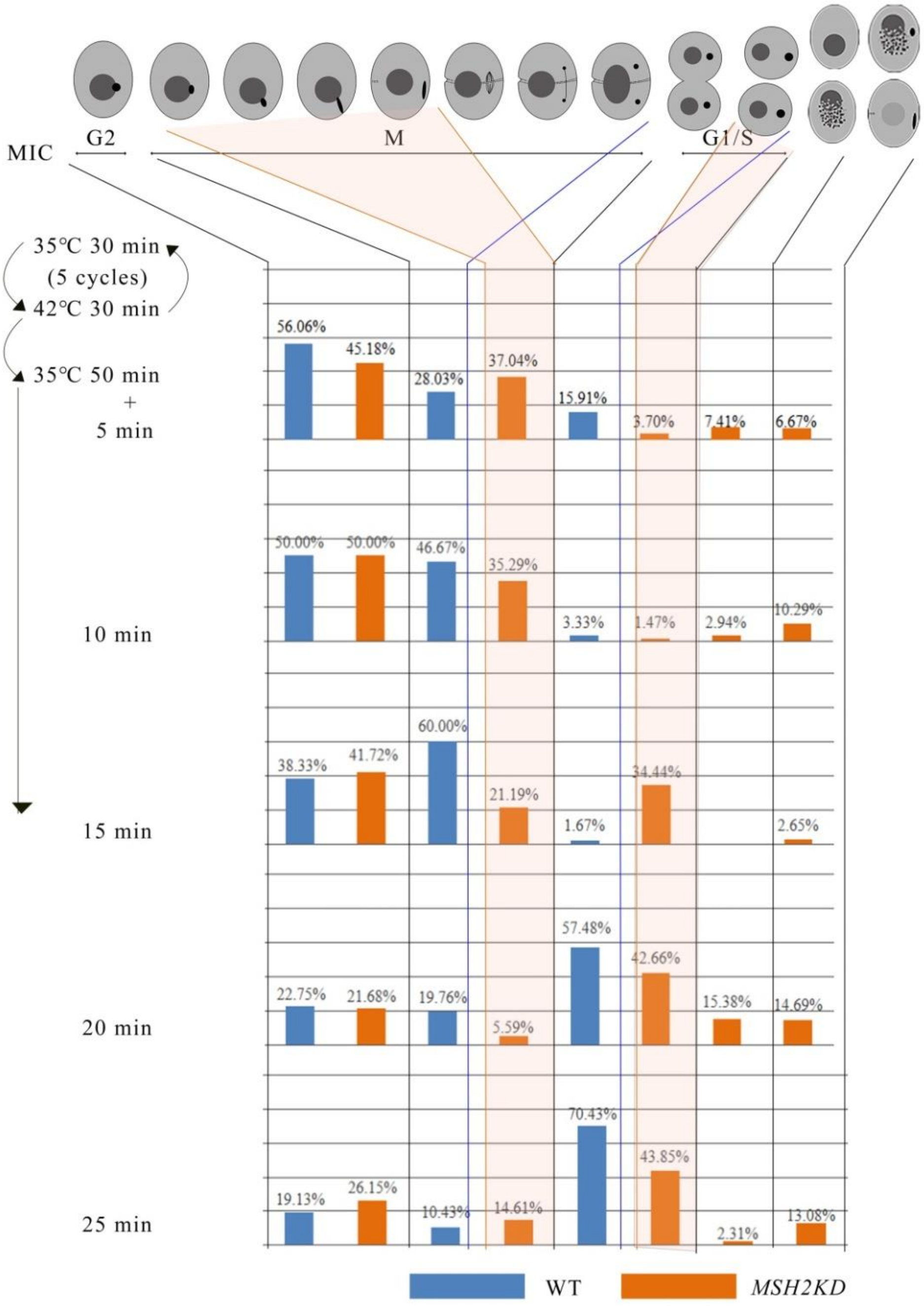

Figure S4. Knockdown of *MSH2* affected nuclear division during vegetative proliferation in *T. thermophila*. Nuclear development statistics of the micronucleus of the *MSH2KD* mutant cell line and wild-type control cells after synchronization during vegetative proliferation. The number of cells counted was greater than 120 cells, for each time point, for each cell line. The topmost part of the diagram shows a diagram of the cell development model.

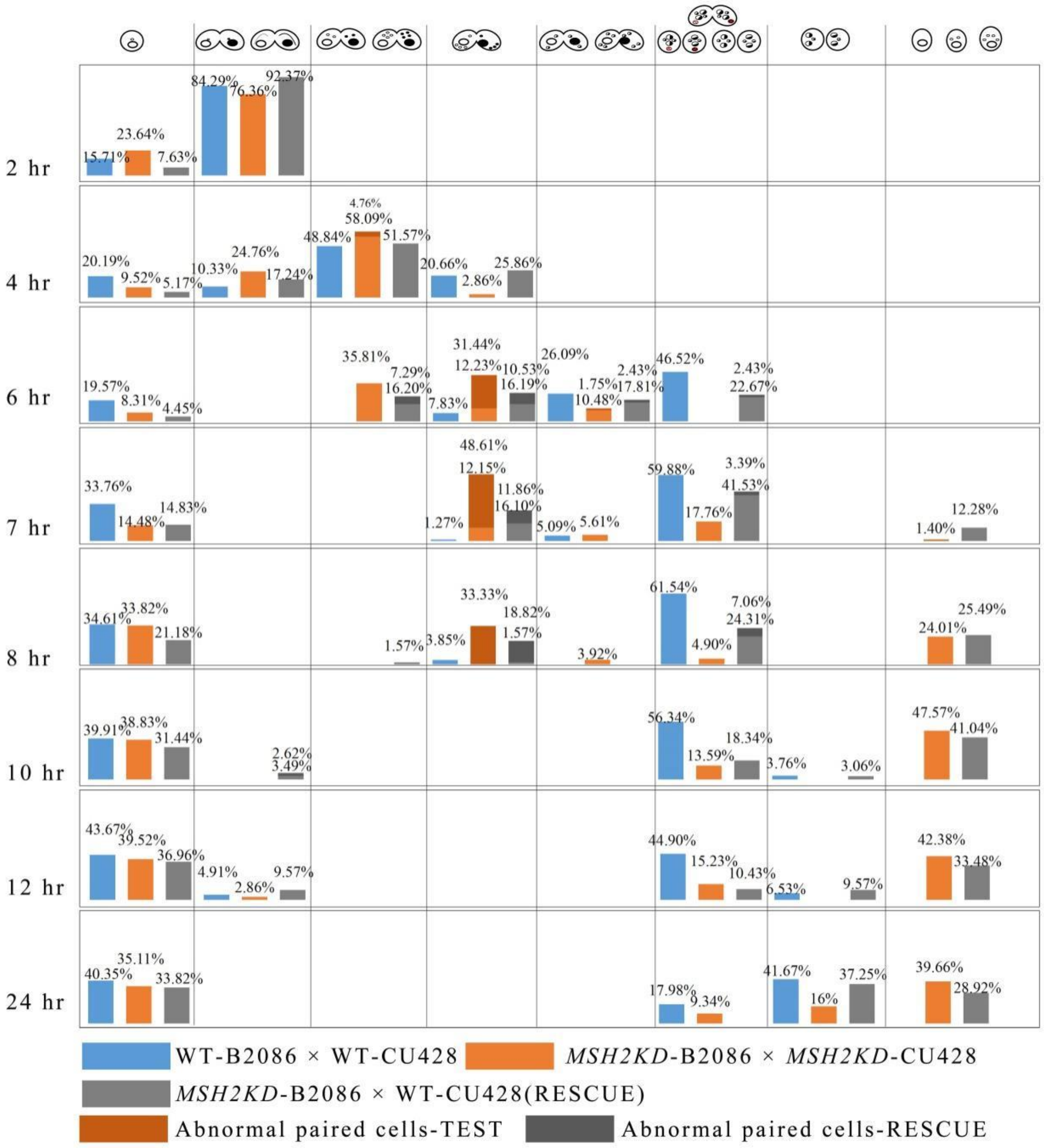

Supplementary Figure S5. Knockdown of *MSH2* affects nuclear division during conjugation in *Tetrahymena*. The number of cells counted was greater than 200 cells, for each time point, for each cell line. The topmost part of the diagram shows a diagram of the cell development model.

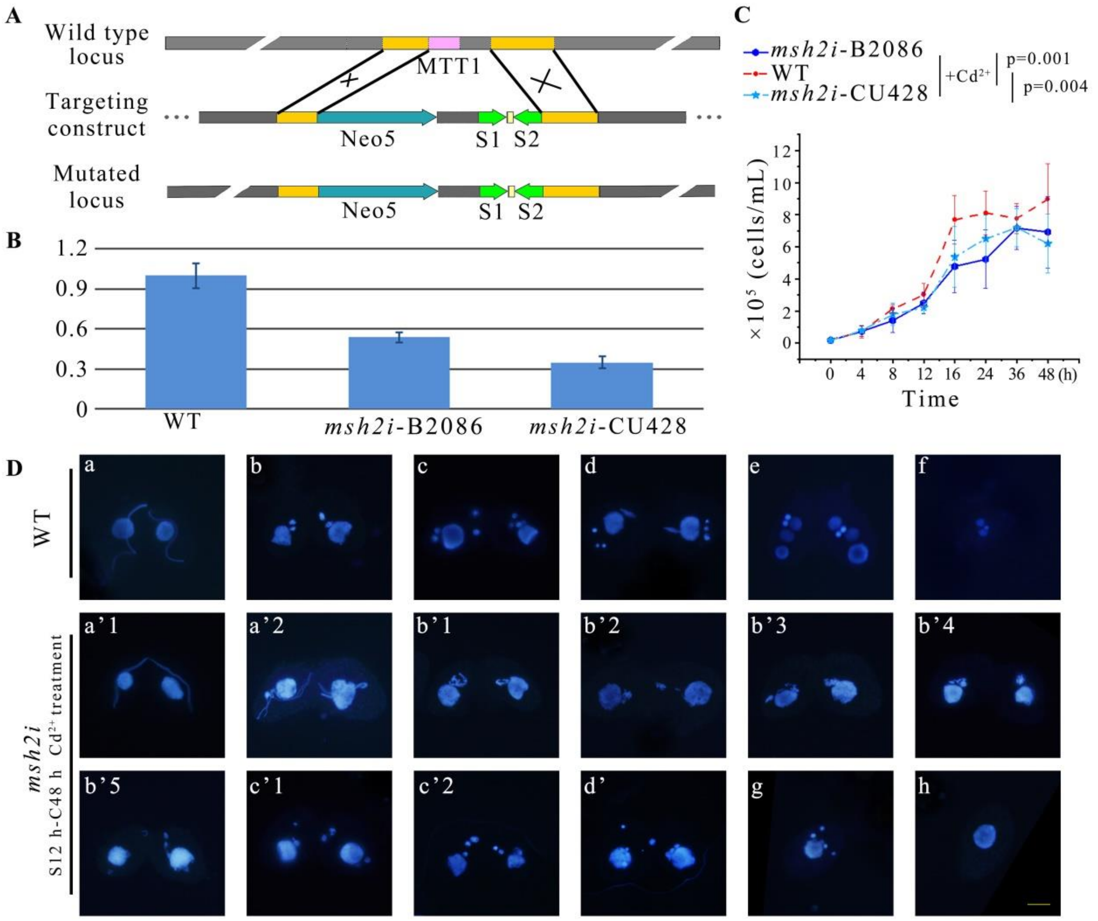

Figure S6. Effect of the knockdown of *MSH2* on nuclear division during conjugation in *Tetrahymena* independent of the accumulation of cellular abnormalities during vegetative proliferation. (**A**) Schematic representation for generating recombinant *msh2i* mutants in *T. thermophile*; (**B**, **C**) *msh2i* cells showed a significant decrease in *MSH2* expression and proliferation capacity; (**D**) Nuclear morphology of *msh2i* mutant and wild-type cells after Cd^{2+} induction which was added at late starvation and during conjunction. DAPI stains the nuclei blue. Scale bar is 10 μm. DAPI stains the nuclei blue.

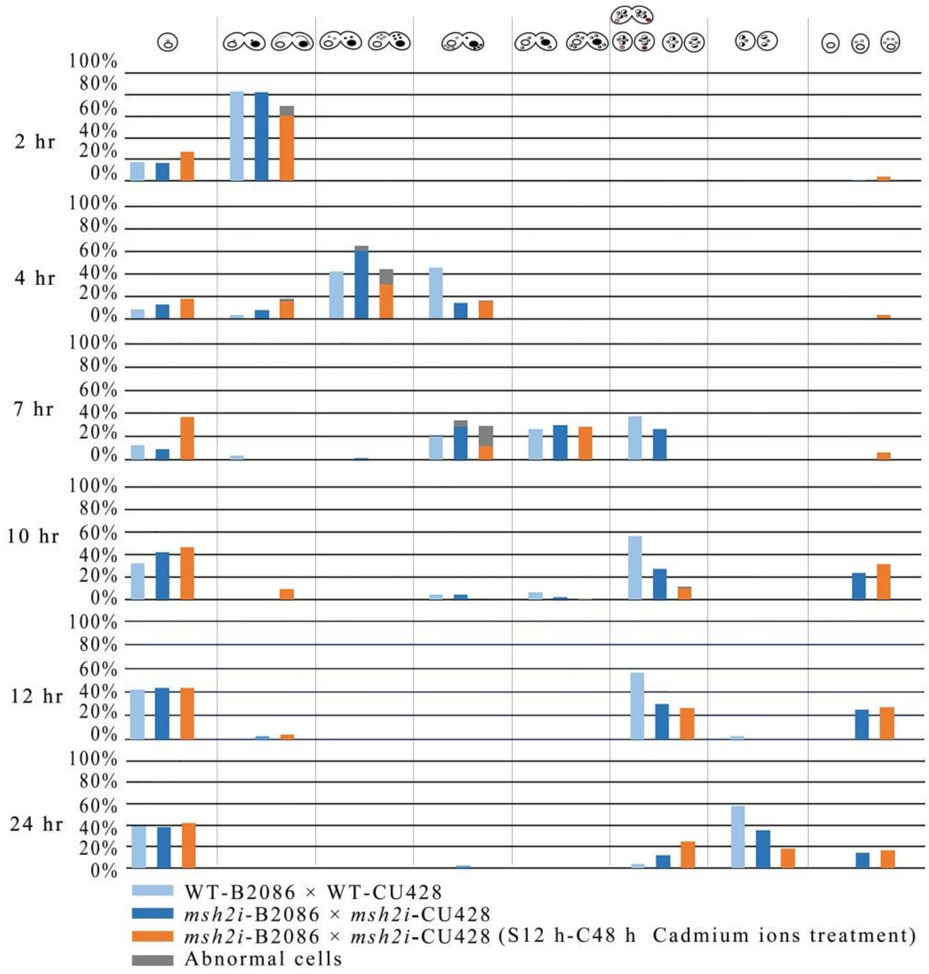

Supplementary Figure S7. Nuclear development statistics of the *msh2i* mutant cell line after Cd^{2+} induction which was added at late starvation and during conjunction. The number of cells counted was greater than 300 cells, for each time point, for each kind of paired cell. The topmost part of the diagram shows a diagram of the cell development model.
